# Laparoscopic Treatment of Placenta Percreta Retention in a Cesarean Scar: A Case Report

**DOI:** 10.3389/fsurg.2014.00006

**Published:** 2014-03-24

**Authors:** Jean-Bernard Dubuisson, Nordine Ben Ali, Jean Bouquet de Jolinière, Manuela Haggenjos, Anis Feki

**Affiliations:** ^1^Hôpital Cantonal de Fribourg, Fribourg, Switzerland

**Keywords:** cesarean section scar, placenta percreta, laparoscopy

## Abstract

Placenta percreta retention within the scar of a previous cesarean section is rare. We report here one of these cases treated successfully by laparoscopy, with uterine repair. Different therapeutic options are described.

## Introduction

Cesarean section is a more and more frequent way delivery. Next to the classical medical indications of cesarean section to avoid complications for the mother and for the fetus, it is getting common to get a cesarean section for convenience because the patient wishes to avoid vaginal delivery. It is the case in many countries and especially in Switzerland. Thankfully, an ectopic pregnancy implanted within a previous cesarean section scar is a rare complication (1). The first reported case was published in 1978 (2): The patient was successfully treated by laparotomy, hysterotomy, pregnancy ablation, cesarean section scar resection, and uterus repair. The incidence is reported to be up to 6.1% of all ectopic pregnancies in women who had at least one cesarean delivery (3). Pregnancies after a cesarean section may be complicated by abnormal adhesion of the placenta in 30–40% of cases (4). Treatment remains controversial and there is no unanimous standard of treatment. Different methods have been reported with variable success rates. Methotrexate is followed by frequent failures. Dilatation and curettage may fail as well, because of the intense trophoblastic invasion of the dehiscent scar with risk of uterine perforation. It is the reason why abdominal surgery may be chosen in selected cases. Mini invasive surgery by laparoscopy allows you to remove the ectopic pregnancy or the percreta placenta and to repair correctly the damaged scar. But ectopic pregnancy in the cesarean section scar may be diagnosed too late, often only when severe life-threatening complications occur such as hemorrhage or uterine rupture. It explains why there were reported cases that needed life-saving hysterectomy (5). The goal of this paper is to report a successful case of an important placenta retention with percreta remnants in the cesarean scar treated by laparoscopy, to discuss the tips and tricks of a laparoscopic treatment according to our experience, and also all the other therapeutic alternatives.

## Materials and Methods

**Figure 1:**
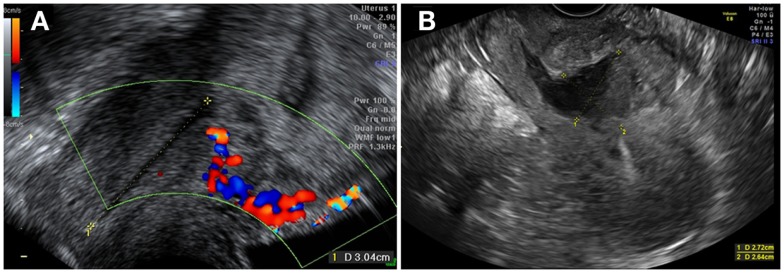
**(A)** Intra-uterine pregnancy at 7 weeks of gestation, with trophoblastic insertion in the cesarean scar site, with a Doppler flow underneath. **(B)** Distended uterine scar after D&C.

We report a case of placenta percreta in the cesarean scar in a 24-year-old patient with a history of cesarean section performed in emergency for fetal distress. One year after the cesarean section, the patient was pregnant. An intra-uterine pregnancy was diagnosed at 7 weeks of amenorrhea (Figure 1A). The low localization in the uterine cavity was confirmed at 8 weeks. Unfortunately, the pregnancy was not evolutive at 10 weeks. The day following the diagnosis, a dilatation, and curettage with aspiration under general anesthesia was performed, with removal of some trophoblastic tissue (Figure 1B). The post-operative course was complicated by uninterrupted moderate bleeding associated with pelvic pain. Three weeks later, because of the persistent symptoms, an ultrasound was performed and a trophoblastic retention (48 mm × 28 mm × 27 mm in size) was diagnosed. Our referent echographer diagnosed the placenta percreta implantation into the cesarean scar. The uterine wall was invisible at the level of the isthmic scar, only the bladder wall was visible. The necessity to perform a laparoscopic treatment was then discussed. The first argument to support laparoscopy was the persistent symptoms with metrorrhagia and abnormal pain. The second argument was the failure of the dilatation and curettage with major trophoblastic retention. The retention was explained by the percreta invasion of the placenta implanted over the scar. The third argument was the thinness of the uterine scar, visible at ultrasound that opened the discussion of a uterine reconstruction. Consequently, the patient was informed of the surgical protocol and also of the possible complications of laparoscopy, especially conversion to laparotomy.

## Results

**Figure 2:**
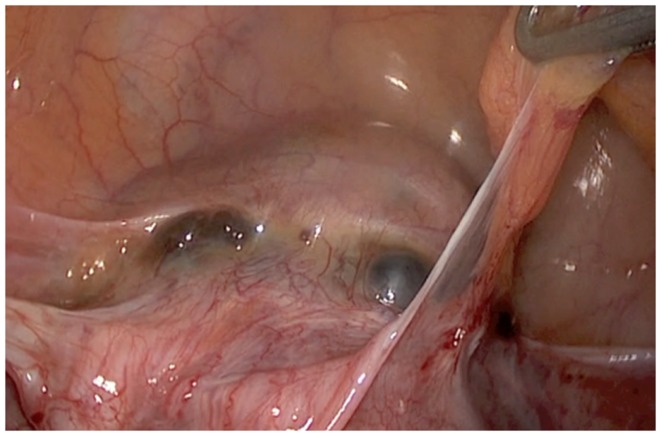
**Laparoscopic aspect of the placenta percreta retention in the cesarean section scar**.

**Figure 3:**
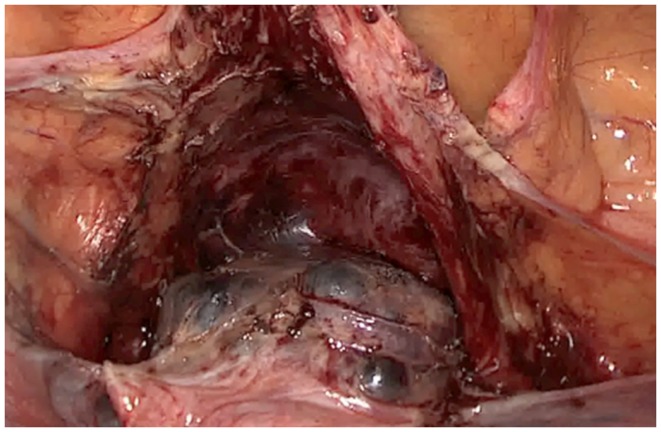
**Vesico uterine cleavage before hysterotomy**.

**Figure 4:**
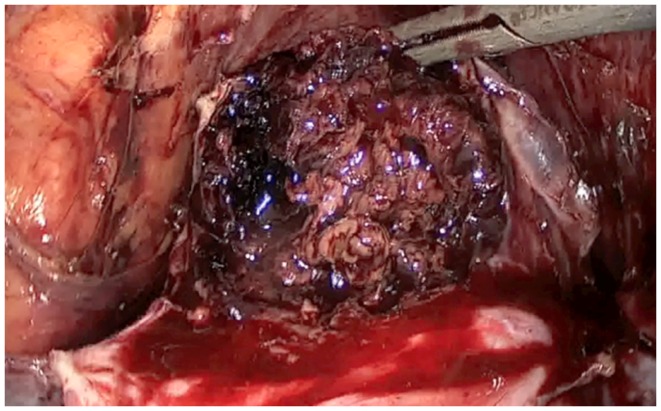
**Ablation of the ectopic pregnancy after hysterotomy**.

**Figure 5:**
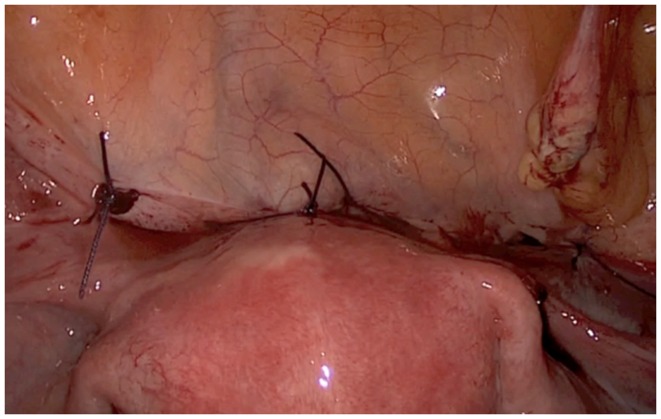
**Final aspect after uterine repair in two layers and reperitonealization**.

The description of the laparoscopic treatment included different steps (Figures 2–5). After CO_2_ insufflation with the Veress needle, a trans-umbilical laparoscopy was performed using a 12 mm trocar. Three 5 mm trocars (supra-pubic and lateral) were inserted. Some post-operative adhesions, close to the anterior wall of the uterus, were visualized and sectioned. The anterior isthmus of the uterus appeared abnormal, presenting a huge hemorrhagic mass of 5 cm in diameter, visible under the serosa and the bladder, with complete dehiscence of the uterine scar, but without intra-peritoneal rupture. The first step of the surgical treatment was the dissection of the mass from the bladder, as low as possible, until healthy uterine tissue. Then, the second step was the incision of the thin uterine wall. The transversal hysterotomy, perpendicular to the long axis of the uterus, was performed a few millimeters above the mass using a monopolar needle with pure section mode and scissors. Because of the bleeding, hemostasis of the upper incisional edge of the hysterotomy was done using bipolar cautery. The cleavage between the thin uterine wall and the huge placenta retention was impossible, confirming the diagnosis of percreta placenta. It is the reason why a second transversal hysterotomy was performed below the mass, in healthy tissue. With this double incision, the mass of trophoblastic retention was detached from the uterus and removed vaginally though the cervical canal. The third step was the uterine repair that was performed immediately to avoid bleeding. The uterine dehiscence was closed with laparoscopic suturing. The hysterorraphy consisted of two layers of separate absorbable sutures of 0 Vicryl (Johnson & Johnson, Hamburg, Germany) followed by a re-approximation of the peritoneum (00 Vicryl). The hemostasis was then controlled. Some trophoblastic fragments were disseminated in the abdominal cavity during the vaginal extraction. They were removed using an endoscopic bag. The general anesthesia duration was 180 min. The duration of post-operative stay was 1 day. A peri-operative drop of 13.6–11.9 g/l of hemoglobin was noted, treated with iron. Myometrial tissue including chorionic villi and clots were observed at histology. The post-operative course was uneventful. Four-months later, the ultrasound showed a good scar healing with a regular line of 4.5 mm thickness. The patient is still not pregnant.

## Discussion

Uterine scar dehiscence after cesarean section may be asymptomatic; the subperitoneal dehiscence is only diagnosed during the next pregnancy or during the cesarean section. In the non-pregnant patient, the dehiscence may be symptomatic with pelvic pain, dysmenorrhea, metrorrhagia, or infertility. The diagnosis of dehiscence of the cesarean scar is usually precised by ultrasound with the evaluation of the thickness of the residual uterine wall. In the event of dehiscence, the residual myometrial thickness is <2.5 mm or the niche is deep, more than 80% of the anterior uterine wall thickness (6). Hysteroscopy is rarely performed after cesarean delivery. It is usually indicated in patients with abnormal uterine bleeding, or postmenstrual spotting. Hysteroscopy shows residual clots or old menstrual blood in an abnormally deep scar. Magnetic resonance imaging (MRI) and hysterography reveal the same anomaly with a large and deep diverticular scar corresponding to the dehiscence of the cesarean scar.

Ectopic pregnancy within the uterine cesarean scar is the rarest form of ectopic pregnancy. It is observed in 1/1800 to 1/2216 pregnancies (4). This localization may be dangerous because trophoblastic invasion of the dehiscent scar may be responsible of massive hemorrhage and uterine rupture (7). Trophoblastic invasion is enhanced when the decidualization of the lower uterine segment is impaired by myometrial disruption. Cesarean section increases fivefold the frequency of future placenta percreta and it is even more frequent with multiple previous cesarean deliveries (8, 9). Currently, there is no consensus and no standard of treatment. Expectant management (4) ends in spontaneous first-trimester miscarriage in 44% of the cases. Expectant management until the third trimester with near-term delivery with a live-born infant is very rare and risky. Herman et al. (10) reported an elective iterative cesarean section at 35 weeks of amenorrhea for severe abdominal pain with the delivery of a healthy baby. But there was a massive blood loss originating from the uterine scar that required hysterectomy and blood transfusion. Because of the high risk of uterine rupture and hemorrhage, it is recommended to interrupt the pregnancy as soon as the diagnosis of ectopic pregnancy can be made without a doubt. In one series, live-threatening hemorrhage was observed in one-third of the cesarean section scar pregnancies (5). Yang (11) reported that 8 out of 11 cases that were treated with a dilatation and curettage were complicated by a severe hemorrhage, necessitating a hysterectomy, or a wedge resection of the uterus.

During the first-trimester of pregnancy, the differential diagnosis may be difficult between intra-uterine spontaneous abortion, cervical-isthmic pregnancy, implantation of placenta percreta remnants in the cesarean scar, and ectopic pregnancy in a cesarean scar. The diagnosis is usually performed by ultrasound.

The ectopic pregnancy in the cesarean section scar is suspected by ultrasound when the uterine cavity and the cervical canal are completely empty and the gestational sac develops in the anterior part of the uterine isthmus and there is no myometrium between the bladder and the sac. Medical treatment shows variable results. Systemic methotrexate may be an option (12). Injection of methotrexate or hyperosmolar glucose into the pregnancy seems more effective (3, 13). But methotrexate treatment may be complicated by secondary hemorrhage (14). In one series, the authors reported 7 cases with severe bleeding out of 17 (11). A success rate of 80% has been published but the resolution of β human chorionic gonadotropin (HCG) may take 6–10 weeks (4) and the regression of the ectopic mass in the scar 2–12 months (13). The success rate seems associated with β HCG levels, with higher risks of complications when the level is exceeding 5000 UI/l.

In cases of placenta percreta remnants implantation in the cesarean scar and in cases of ectopic pregnancy, dilatation, and curettage under ultrasound guidance is also a therapeutic option, but the risk of failure is quite high. In fact, the niche of the scar, where the trophoblastic tissue is implanted, is not in the axis of the rigid curette. Perforation of the cesarean scar may also occur (11). Methotrexate treatment after dilatation and curettage may be used to treat the residual percreta retention in this localization (15). But a relatively slow decline of β HCG levels and a potential risk of massive bleeding and uterine rupture have been described (16). A prospective controlled trial, comparing uterine artery embolization (UAE) followed by suction curettage (UAE group) and methotrexate (MTX group) followed by suction curettage for the management of pregnancy implanted within a cesarean scar showed that UAE group had much less bleeding, shorter stay duration and lower hysterectomy rate than MTX group (11).

The main drawback of conservative treatment is that they do not treat the pathologic residual scar associated with diverticular and with dehiscence. This problem may be discussed when the patient wants another pregnancy, because of the theoretical risk of uterine rupture during following pregnancies. But there are no statistics concerning this precise risk. We have also to mention other symptoms to treat such as pelvic pain and bleeding. All these considerations make surgical treatment a possible option, especially operative laparoscopy. The first laparoscopic management of an ectopic pregnancy in a previous cesarean section scar was described by Lee (17). Other authors (6) described the laparoscopic repair of the scar dehiscence after cesarean section. In our case report of laparoscopic treatment, several points may be discussed. First, we considered the initial diagnosis of miscarriage, the failure of treatment by curettage, and the diagnosis of placenta percreta remnants in the cesarean scar. Unfortunately, this last diagnosis was missed by the first gynecologist. So, during the first-trimester of pregnancy, in a patient with past history of cesarean section, it is important to eliminate by ultrasound an ectopic implantation or a placenta percreta in the scar. Our second concern was the management by operative laparoscopy. It is contra-indicated if the patient is admitted for severe life-threatening hemorrhage. In such cases, an emergency laparotomy is mandatory. In other cases, laparoscopy may be an option because it treats the actual problem with the removal of placenta percreta retention and it also allows resection of the scar and repair of the uterus. The procedure may be difficult in some cases. The bladder may be adherent to the trophoblastic tissue and to the uterus. Usually, the cleavage is possible, starting laterally, close to the round ligaments. The hemorrhagic risk, that can be suspected by the Doppler ultrasound and high levels of β HCG (>15,000 IU/l) may be prevented with uterine artery occlusion (clip or bipolar cautery) at the beginning of the procedure (18). Finally, the repair must be done carefully (19). Hemostasis of the bleeding vessels of the healthy edges is necessary to avoid post-operative hematomas. A suture with two layers of interrupted absorbable stitches may help a favorable healing. A check-up with imaging 2–4 months after surgery can assess the quality of the repair. We aim for a linear and regular scar with a significant thickness of more than 2.5 cm (6). But it is not proven by any data that the uterus repair is of clinical benefice in further pregnancy. Long series are needed to objectively evaluate the options to avoid complications such as hemorrhage and uterine rupture (20, 21). The rational to treat laparoscopically the placenta percreta retention in the cesarean scar has to be demonstrated. The publication of case reports may bring discussion about best therapeutic alternatives.

## Conflict of Interest Statement

The authors declare that the research was conducted in the absence of any commercial or financial relationships that could be construed as a potential conflict of interest.
